# Retrospective analysis of the quality of reports by author-suggested and non-author-suggested reviewers in journals operating on open or single-blind peer review models

**DOI:** 10.1136/bmjopen-2015-008707

**Published:** 2015-09-29

**Authors:** Maria K Kowalczuk, Frank Dudbridge, Shreeya Nanda, Stephanie L Harriman, Jigisha Patel, Elizabeth C Moylan

**Affiliations:** 1 BioMed Central, London, UK; 2 Department of Non-communicable Disease Epidemiology, London School of Hygiene and Tropical Medicine, London, UK; 3 Springer Healthcare, London, UK

**Keywords:** Peer review, Journalology, Review Quality Instrument

## Abstract

**Objectives:**

To assess whether reports from reviewers recommended by authors show a bias in quality and recommendation for editorial decision, compared with reviewers suggested by other parties, and whether reviewer reports for journals operating on open or single-blind peer review models differ with regard to report quality and reviewer recommendations.

**Design:**

Retrospective analysis of the quality of reviewer reports using an established Review Quality Instrument, and analysis of reviewer recommendations and author satisfaction surveys.

**Setting:**

BioMed Central biology and medical journals. *BMC Infectious Diseases* and *BMC Microbiology* are similar in size, rejection rates, impact factors and editorial processes, but the former uses open peer review while the latter uses single-blind peer review. The *Journal of Inflammation* has operated under both peer review models.

**Sample:**

Two hundred reviewer reports submitted to *BMC Infectious Diseases*, 200 reviewer reports submitted to *BMC Microbiology* and 400 reviewer reports submitted to the *Journal of Inflammation*.

**Results:**

For each journal, author-suggested reviewers provided reports of comparable quality to non-author-suggested reviewers, but were significantly more likely to recommend acceptance, irrespective of the peer review model (p<0.0001 for *BMC Infectious Diseases, BMC Microbiology* and the *Journal of Inflammation*). For *BMC Infectious Diseases*, the overall quality of reviewer reports measured by the Review Quality Instrument was 5% higher than for *BMC Microbiology* (p=0.042). For the *Journal of Inflammation*, the quality of reports was the same irrespective of the peer review model used.

**Conclusions:**

Reviewers suggested by authors provide reports of comparable quality to non-author-suggested reviewers, but are significantly more likely to recommend acceptance. Open peer review reports for *BMC Infectious Diseases* were of higher quality than single-blind reports for *BMC Microbiology.* There was no difference in quality of peer review in the *Journal of Inflammation* under open peer review compared with single blind.

**Strengths and limitations of this study**

- This is the first study to compare quality of peer review between two journals that are very similar in most aspects but differ in peer review model (*BMC Microbiology* operates single-blind peer review while *BMC Infectious Diseases* operates open peer review).
- It is the first study to analyse the effect of change in peer review model across a single journal (*Journal of Inflammation*).
- A large number of reviewer reports were analysed (800), resulting in a well-powered study in three different journals publishing research in both biology and medicine.
- We observed moderate agreement between raters for peer review report quality, although the raters did not discuss the rating for *BMC Infectious Diseases* and *BMC Microbiology*, and did not confer about their ratings for individual reports for any journal.
- We had to use a different study design for the *Journal of Inflammation* section of the study than that used for the *BMC Infectious Diseases* and *BMC Microbiology* section, due to insufficient number of manuscripts fulfilling the inclusion criteria.

## Introduction

Most scholarly journals operate one of three types of peer review models: single blind, where the reviewers know the identity of the authors but not vice versa; open peer review, where authors and reviewers both know each other's identity, or double-blind peer review where the author and reviewer names are both blinded. Some journals publish reviewer reports (signed or anonymous) and authors’ responses together with accepted articles. Many journals allow, or in some cases require, authors who submit manuscripts to suggest potential reviewers. This information is available to editors who are responsible for selecting appropriate reviewers.

Previous studies have compared quality of reviewer reports under open and single-blind peer review across various journals,^1^ and analysed open peer review,^2^ public peer review,^3^ the proportion of authors who suggested reviewers^4^ and differences between recommendations by reviewers who were either suggested or excluded by the authors.^5^ It has been found that the reviewers chosen by editors are statistically-significantly more critical than those suggested by the authors.^1^ ^3–9^ Intriguingly, the majority of the studies have been conducted on medical journals and there are no studies on biology journals.

The objective of this study was to assess whether the reviewers suggested by the authors are biased in their assessment of a manuscript, and whether the quality of their reports is different from reports prepared by reviewers suggested by editors. A second objective was to compare open peer review with single-blind peer review. This study was the first to analyse a large number of reviewer reports (800 in total) in both medical and biology journals, and compare quality of reviewer reports between open and single-blind peer review models and between author-suggested and non-author-suggested reviewers.

The initial findings from this research were presented in poster format at the 7th Peer Review Congress in Chicago in 2013. The poster has been deposited on F1000 Posters.^10^

## Methods

### Analysed journals

We analysed peer review reports for manuscripts submitted in 2010–2011 to *BMC Microbiology*, which operates single-blind peer review, and *BMC Infectious Diseases*, which operates open peer review. *BMC Microbiology* and *BMC Infectious Diseases* were launched in 2000 as part of the BMC series of journals.^11^ All policies and processes are the same across the series of journals. *BMC Microbiology* and *BMC Infectious Diseases* are similar in terms of size, impact factors and rejection rates, and cover similar subject areas (table 1). The journals are managed by a team of in-house editors who work very closely with their editorial boards. Manuscripts are handled by academic associate editors and section editors, who select and check all invited reviewers. They may decide to use the reviewers suggested by the authors, by other reviewers, or by BioMed Central's PubMed search tool, or select reviewers based on their own knowledge and searches. The reviewers receive the same invitations to review and the same templates to prepare their reports. The only difference is that for *BMC Infectious Diseases* reviewers must agree to open peer review, including the publication of their signed reports if the manuscript is accepted.

**Table 1:** Information about *BMC Microbiology* and *BMC Infectious Diseases* in the period covered by this research

	*BMC Microbiology*	*BMC Infectious Diseases*
Impact factor 2012	3.10	3.03
Number of articles published in 2012	307	386
Rejected submissions in 2010 and 2011, %	52.5	55.5
Peer review model	Single blind	Open

The third analysed journal was the *Journal of Inflammation*, which was launched in 2004. It originally operated under an open peer review model, but adopted a single-blind peer review policy on 29 January 2010 (table 2). The study period was from 2007–2011.

**Table 2:** The *Journal of Inflammation* in the period covered by this research

*Journal of Inflammation*	2007–2009 (open peer review)	2010–2011 (single-blind peer review)
Impact factor	None	2.017–2.263
Number of articles published	58	150
Rejected submissions, %	33	50

### Selection of reviewer reports

For each journal, we analysed reviewer reports on manuscripts presenting original research that had a final decision (accept or reject). In each of *BMC Microbiology* and *BMC Infectious Diseases*, we identified 100 manuscripts that had two reviewers each, one suggested by the authors and one by another party (BioMed Central's PubMed search tool comparing the abstract of the manuscript to abstracts in PubMed, another reviewer or editor). This was achieved by searching the journal's database for consecutive submissions in the analysed period 2010–2011. The *Journal of Inflammation* published fewer original research articles and a larger proportion of review articles and case reports that were not suitable for our analysis. In the *Journal of Inflammation*, we analysed 200 reviewer reports for research manuscripts submitted consecutively counting back from the date of change of peer review model (under open peer review), and 200 counting forward (under single-blind peer review). There was an insufficient number of research article submissions that had two reviewers, one suggested by the authors and one by another party. We analysed 193 manuscripts, in total, that had between one and five reviewers. As a result, for the *Journal of Inflammation*, we analysed reports spanning a longer period of time (2007–2011), and the number of reports provided by author-suggested reviewers was not equal to that provided by non-author-suggested reviewers (table 3).

**Table 3:** Numbers of reports analysed in each journal

	Open peer review		Single-blind peer review	
***BMC Microbiology*** 100 manuscripts analysed	NA		100 reports from reviewers nominated by **authors**	100 reports from reviewers **not** nominated by authors
***BMC Infectious Diseases*** 100 manuscripts analysed	100 reports from reviewers nominated by **authors**	100 reports from reviewers **not** nominated by authors	NA	
***Journal of Inflammation*** 193 manuscripts analysed	50 reports from reviewers nominated by **authors**	150 reports from reviewers **not** nominated by authors	29 reports from reviewers nominated by **authors**	171 reports from reviewers **not** nominated by authors

NA, not applicable.

### Assessing quality of reviewer reports

Each peer review report was rated using an established Review Quality Instrument (RQI)^12^ (table 4). Each report was rated separately and independently by two senior members of the editorial staff at BioMed Central. The peer review model and whether the reviewer was author suggested was unknown to the raters. However, the raters were not blinded to the reviewers’ identity. When rating the quality of reports for *BMC Microbiology* and *BMC Infectious Diseases*, formal discussions were not held between the raters on how to use the RQI. However, before analysing reports for the *Journal of Inflammation*, the raters agreed on the criteria for each score on the RQI before rating the peer review reports (see Discussion section).

**Table 4:** Reproduction of the Review Quality Instrument (RQI).^12^

RQI				
**Q1. Did the reviewer discuss the importance of the research question?**				
1	2	3	4	5
Not at all				Discussed extensively
**Q2. Did the reviewer discuss the originality of the paper?**				
1	2	3	4	5
Not at all				Discussed extensively with references
**Q3. Did the reviewer clearly identify the strengths and weaknesses of the method (study design, data collection and data analysis)?**				
1	2	3	4	5
Not at all				Comprehensive
**Q4. Did the reviewer make specific useful comments on the writing, organisation, tables and figures of the manuscript?**				
1	2	3	4	5
Not at all				Extensive
**Q5. Were the reviewer’s comments constructive?**				
1	2	3	4	5
Not at all				Very constructive
**Q6. Did the reviewer supply appropriate evidence using examples from the paper to substantiate their comments?**				
1	2	3	4	5
None substantiated		Some substantiated		All substantiated
**Q7. Did the reviewer comment on the author’s interpretation of the results?**				
1	2	3	4	5
Not at all				Discussed extensively
**Q8. How would you rate the tone of the review?**				
1	2	3	4	5
Abusive				Courteous

Reproduced with permission, License Number: 3617630208550.

### Analysis of reviewer recommendations

For the journals studied, peer reviewers could choose one of six recommendations suggested in the reviewer form, or choose not to provide recommendation. To facilitate the analysis, we grouped these recommendations into four categories (table 5).

**Table 5:** Grouping of reviewer recommendations for the purpose of our analysis

Recommendations provided in the reviewer form	Recommendations grouped for our analysis
Accept without revision	Accept
Accept after discretionary revisions	
Accept after minor essential revisions	
Unable to decide on acceptance or rejection until the authors have responded to the major compulsory revisions	Revise
Reject because too small an advance to publish	Reject
Reject because scientifically unsound	
No recommendation	No recommendation

### Author surveys

All corresponding authors were asked to complete author surveys after publication of their article (see online supplementary material). The survey consists of 18 questions related to different aspects of the editorial and production processes, of which one is, ‘How helpful were the reviewers’ comments?’. The authors were asked to score their answers to the questions on a scale from 1 (very poor) to 5 (very good). We compared the authors’ ratings for the question on the helpfulness of peer review comments compared to scores for other questions.

### Statistical analyses

It was previously shown^1^ that a total of 110 papers gives 90% power at p<0.05 to detect a difference in RQI of one-half its SD; we therefore considered our sample sizes to be well powered.

Inter-rater agreement was measured by weighted κ with quadratic weights. For each article and reviewer, the mean of the two rater scores was then used in subsequent analyses. Review quality was compared between open and single-blind review models using the unpaired Mann-Whitney U test. Review quality was compared between author suggested and other reviewers, using the paired Mann-Whitney U test in *BMC Microbiology* and *BMC Infectious Diseases* (in which each article had one reviewer of each type), and using the unpaired test in the *Journal of Inflammation*. Bonferroni correction was applied for eight tests in each journal.

To test whether author-suggested reviewers were more positive than other reviewers, we coded reviewer recommendations as reject=0, revise=1, or accept=2, and treated this value as the response in linear regression with reviewer type (author suggested or other) as the predictor. Interaction terms with review model (open or single blind) were included in exploratory analysis but dropped if found to be non-significant.

Association of reviewer recommendations with the review model was tested in logistic regression with review model (open or single blind) as the response, and reviewer recommendation coded as a three-level categorical predictor (accept, revise, reject).

Association of reviewer recommendations with the final decision was tested in logistic regression with the final decision as the response and reviewer recommendation coded as a three-level categorical predictor (accept, revise, reject) with different effects for author-suggested and non-author-suggested reviewers. Interaction terms with review model (open or single blind) were also included in exploratory analysis but dropped if found to be non-significant.

In all regression analyses, each review was treated as one observation and cluster SEs used to allow for multiple reviews of each paper.

Responses to author surveys were tested using the two-sample Mann-Whitney U test.

All analyses were conducted in R.

## Results

### Analysed journals and reports

The analysed journals and reports are shown in tables 1–3.

### Quality of reviewer reports

#### Rater agreement on quality of reports

We found moderate agreement between the raters for *BMC Microbiology* and *BMC Infectious Diseases*, with weighted κ values generally around 0.4 or higher. For the *Journal of Inflammation*, where the raters agreed on the criteria for each score of the RQI before rating the reports, the agreement was stronger for six of eight questions, but still in a moderate range (see online supplementary table S1). Our agreements were lower than found in previous studies using the RQI,^12^ ^13^ which might be due to the particular subject matter of the reviewed articles or to bias in the raters’ perception on what they deemed important as a review comment.

#### Quality of reviewer reports on open and single-blind peer review

**Figure 1:**
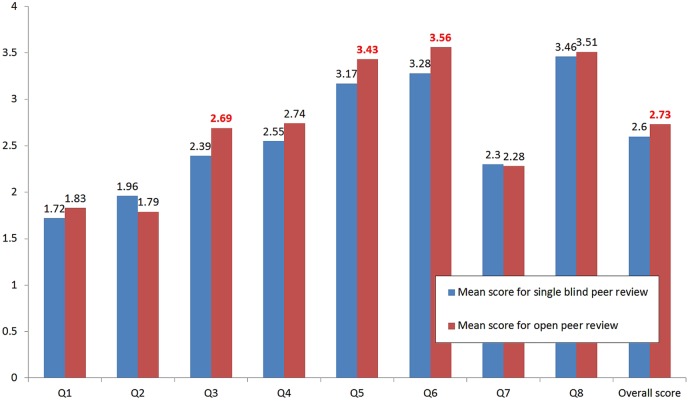
Comparison of Review Quality Instrument scores between ***BMC Infectious Diseases*** (open peer review) and ***BMC Microbiology*** (single-blind peer review). Three questions obtained statistically significantly higher scores in *BMC Infectious Diseases*: Q3: Did the reviewer clearly identify the strengths and weaknesses of the method (study design, data collection and data analysis)? (p=0.004) Q5: Were the reviewer’s comments constructive? (p=0.0046) Q6: Did the reviewer supply appropriate evidence using examples from the paper to substantiate their comments? (p=0.0015) This led to a 5% improvement of the overall score (p=0.042). Values in bold red denote p<0.05.

For questions 3 (strengths and weaknesses of the methods), 5 (constructiveness) and 6 (supplying appropriate evidence to substantiate comments) of the RQI, there was a significantly higher score for *BMC Infectious Diseases* (open peer review) compared with *BMC Microbiology* (single-blind peer review; figure 1). This led to a 5% improvement of the overall score (p=0.042 averaging both raters); however, this was significant for one rater (p=0.02) but not the other (p=0.39), so may not represent a genuine difference.

For the *Journal of Inflammation*, however, no significant differences were seen in review quality between open and single-blind models (see online supplementary table S2).

#### Quality of reports provided by author-suggested and non-author-suggested reviewers

There is nominally significant evidence that non-author-suggested reviewers obtained higher scores on Q6 (how well the reviewer substantiated their comments) and Q7 (comments on the author’s interpretation of results) in *BMC Infectious Diseases* (open peer review) than the scores obtained by reviewers in *BMC Microbiology* (single-blind peer review). However, these differences are not significant when adjusted for multiple testing. No other questions showed significant differences between author-suggested and non-author-suggested reviewers (see online supplementary tables S3 and S4).

In the *Journal of Inflammation*, a nominally significant difference in review quality was seen for Q3 (comments on strengths and weaknesses of the method), but this was not significant after correction for multiple testing. No other significant differences were seen in review quality between author suggested and other reviewers (see online supplementary table S5).

### Reviewer recommendations to accept or reject

**Figure 2:**
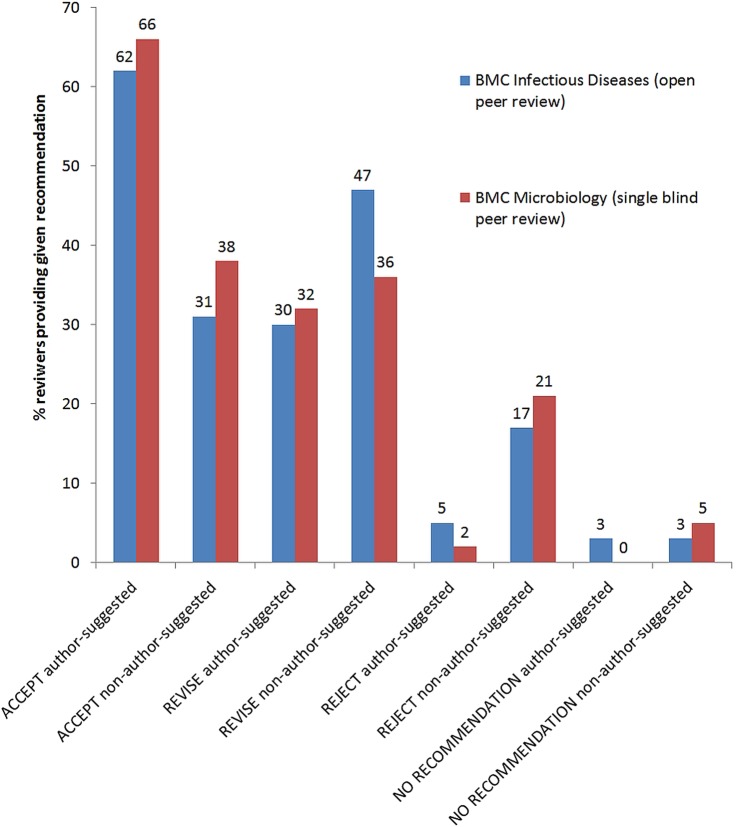
Percentage of reviewers providing a given recommendation for the manuscripts in *BMC Infectious Diseases* and *BMC Microbiology*.

In their initial report, most of the reviewers provided a recommendation to either accept or reject the manuscript. For *BMC Infectious Diseases* (open peer review) and *BMC Microbiology* (single-blind peer review) the numbers of reviewer recommendations of each kind were similar, suggesting a lack of difference between open and single-blind peer review models (figure 2). In both journals, author-suggested reviewers were more positive compared with non-author-suggested reviewers (*BMC Infectious Diseases* p=1.4×10^−6^; *BMC Microbiology* p=6.3×10^−8^). About two-thirds of author-suggested reviewers recommend acceptance and only 2–5% recommended rejection (see figure 2).

**Figure 3:**
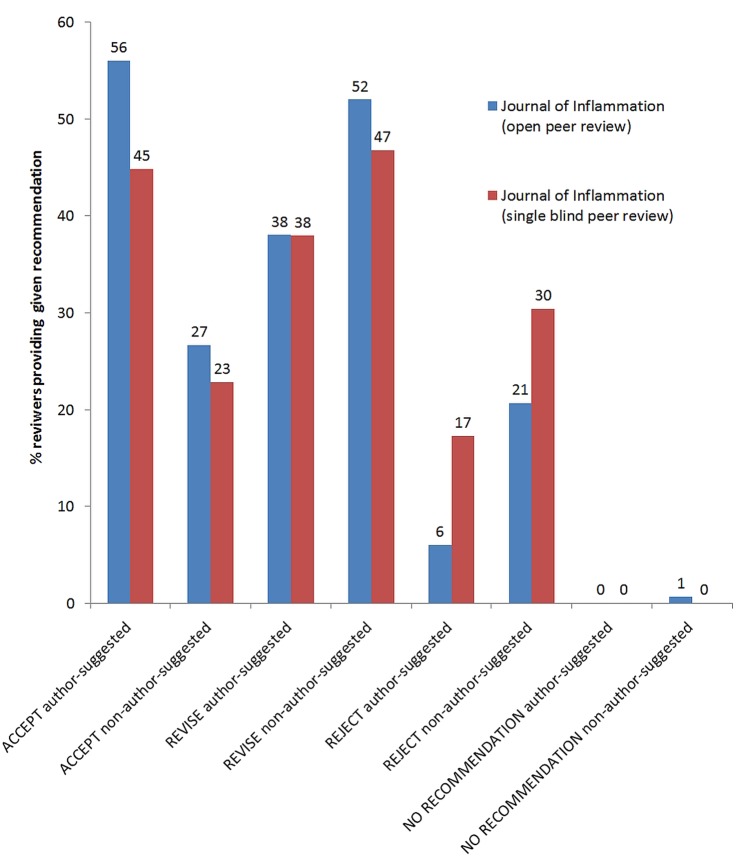
Percentage of reviewers providing a given recommendation for the manuscripts in *Journal of Inflammation*.

For the *Journal of Inflammation*, author-suggested reviewers also returned significantly more favourable recommendations than other reviewers (p=3×10^−6^). Again, there was no significant difference between open and single-blind review models (figure 3).

We found 13 manuscripts in *BMC Infectious Diseases* and 17 in *BMC Microbiology*, where the author-suggested reviewer recommended acceptance or revisions, while the non-author-suggested reviewer recommended rejection and the final outcome was rejection. However, there were only two accepted manuscripts in *BMC Infectious Diseases* and four accepted in *BMC Microbiology* where the author-suggested reviewer recommended acceptance, and the non-author-suggested reviewer recommended rejection. Recommendations of both types of reviewers predicted the acceptance or rejection of the manuscript, but the view of the non-author-suggested reviewer better predicted the final outcome (p=7.3×10^−19^) compared with the author-suggested reviewer (p=1.3×10^−5^). There were no differences between *BMC Infectious Diseases* and *BMC Microbiology* in this respect. However, in the *Journal of Inflammation*, there was no significant difference between author-suggested and non-author-suggested reviewers in predicting the final decision.

### Author surveys

We analysed all author surveys that were available at the end of September 2013: 741 surveys for *BMC Microbiology* (single-blind peer review), 685 for *BMC Infectious Diseases* (open peer review) and 49 (open peer review) and 47 (single-blind peer review) for the *Journal of Inflammation*.

Authors in *BMC Infectious Diseases* felt that reviewer comments were more helpful than did the authors in *BMC Microbiology* (p<0.0001, see online supplementary table S6). However, most other questions relating to managing other editorial and production processes on the manuscript tended to be scored more favourably (p<0.05) as well.

For the *Journal of Inflammation*, we did not find statistically significant differences between surveys returned by authors of manuscripts that underwent open or single-blind peer review, for any question regarding the editorial and production processes.

## Discussion

The peer review process in journals has been criticised for being slow, inconsistent and biased.^14^ However, there is a paucity of studies investigating how peer review actually works, including the quality of reviewer reports, and differences between peer review models and types of reviewers. We were able to compare quality of peer review between *BMC Infectious Diseases* (open peer review) and *BMC Microbiology* (single-blind peer review), two journals that differ in peer review model but are otherwise very similar. We also assessed the effect of change in peer review model within a single journal, the *Journal of Inflammation*, which has changed its peer review model. It gave us unique opportunity to assess the impact of the peer review model on the quality of peer review and recommendations made by the reviewers. In the analysed journals, we were also able to compare the quality of reports and recommendations provided by reviewers suggested by the authors, compared with reviewers suggested by other parties (ie, BioMed Central's PubMed search tool comparing the abstract of the manuscript to abstracts in PubMed, other reviewers and editors). The large sample size of analysed reports ensured statistically robust results.

The main findings are that the quality of peer review reports was slightly higher in *BMC Infectious Diseases* (open peer review) compared with *BMC Microbiology* (single-blind peer review), but we did not find a difference for the open versus single-blind review in the *Journal of Inflammation*. These results suggest that it may be advantageous to use open peer review but they do not undermine the validity of using the single-blind approach.

In none of the three analysed journals did we find a difference in *quality* of peer reviewer reports written by author-suggested reviewers compared with those written by reviewers suggested by other parties, but in all three journals the reviewers suggested by the authors were much less likely to recommend rejection and more likely to recommend acceptance than reviewers suggested by other parties. Overall reviewer recommendations were similar in *BMC Infectious Diseases* compared with *BMC Microbiology*, suggesting no difference between open and single-blind peer review. However, in the *Journal of Inflammation*, we found that reviewers were more likely to recommend acceptance under open peer review as compared with under single-blind peer review. Author satisfaction was higher for *BMC Infectious Diseases* compared with *BMC Microbiology*, including the response to the question regarding the helpfulness of comments from peer reviewers. In *the Journal of Inflammation*, there was no difference in author satisfaction between the periods of time when the journal operated on open or single-blind peer review.

### Quality of reviewer reports

The single-blind peer review model is the most common model of peer review used in journals in the field of biology and medicine. The *BMJ* was one of the first journals to open up peer review in 1999.^15^ This decision was based on the results of a randomised controlled trial^2^ where the quality of reviewer reports was assessed using the same RQI^12^ that we used in the current study. Similar to our analysis, the *BMJ* study found no significant differences between the anonymous and non-anonymous reviewers with regard to the quality of their reports.^2^

Another study of open peer review, using a modified version of the RQI,^12^ was conducted at the medical journal, the *British Journal of Psychiatry.*^13^ Interestingly, that study found that reports under open peer review were of 5.5% higher quality than unsigned reports, which is consistent with the 5% difference we found between scores for reviewer reports in *BMC Microbiology* (single-blind peer review) and in *BMC Infectious Diseases* (open peer review). Moreover, the signed reviewer reports scored significantly higher on the same two questions as our open reports for *BMC Infectious Diseases* (Q3 regarding methodology and Q5 regarding constructiveness of comments). It was also very encouraging to see that, of the 322 reviewers, as many as 245 (76%) agreed to sign their reports for the *British Journal of Psychiatry.*^13^

Under open peer review, reviewers are more accountable for their reviews, which may account for the higher scores we observed for Q3 (discussing the strengths and weaknesses of the method), Q5 (the more constructive reports) and Q6 (supplying appropriate evidence to substantiate the comments), which led to a 5% improvement of the overall score (p=0.042).

There is another possible explanation for the higher score that reviewer reports obtained on open peer review for Question 3 (whether the reviewer clearly identified the strengths and weaknesses of the method, study design, data collection and data analysis). Medical research follows several distinct types of defined study designs (see The EQUATOR Network^16^ for more information). It is therefore possible that, in evaluating medical research as compared with evaluating biology manuscripts, reviewers are more likely to comment on whether the authors used an appropriate study design and appropriate methodology, as there are existing reporting guidelines to follow. We found that key words such as ‘study design’, ‘guideline(s)’, ‘methodology’ and ‘data analysis’ occurred more frequently in reviewer reports for *BMC Infectious Diseases* compared to reviewer reports for *BMC Microbiology*, although it did not reach statistical significance (Fisher exact test, p=0.2).

In the current study, we have not found significant differences in the quality of reports provided by author-suggested reviewers compared with non-author-suggested reviewers. This has also been noted in previous studies.^1^ ^9^

### Reviewer recommendations to accept or reject

Author-suggested reviewers tend to provide more favourable recommendations than other types of reviewers. This result is consistent for the three journals we analysed, irrespective of the peer review model. This is also consistent with previous studies on this topic.^1^ ^3–9^ However, in both *BMC Infectious Diseases* (open peer review) and *BMC Microbiology* (single-blind peer review), recommendations made by non-author-suggested reviewers were a better predictor of the final decision to accept or reject the manuscript than recommendations made by author-suggested reviewers. It seems that these reviews carried more weight with journal editors (unconsciously or consciously).

In the *Journal of Inflammation*, we found that reviewers provided significantly more favourable recommendations under open peer review. This effect was found in some of the previous studies^1^ ^13^ but not all.^2^ Also, acceptance rates were significantly higher under open peer review than on single-blind peer review (67% vs 50%, see table 3). However, it is difficult to determine if this effect is due to the peer review model or other factors. The *Journal of Inflammation* is smaller than *BMC Microbiology* or *BMC Infectious Diseases*, and its analysed reports span a longer period of time, from September 2007 (shortly after the journal was launched) to September 2011. The change in peer review model was also accompanied by a change in editorship and coincided with the journal receiving its first impact factor. It is possible that these factors impacted on how the peer reviewers were selected, and how they perceived the journal and made recommendations.

### Author satisfaction

Authors on *BMC Infectious Diseases* (open peer review) gave slightly higher scores for the question on the helpfulness of reviewers’ comments, compared with *BMC Microbiology* authors (single-blind peer review). However, they also gave higher scores for every other question, so it may be the case that the overall editorial process was perceived to be better at *BMC Infectious Diseases*, or that it simply reflects different priorities among research fields. The score for the *Journal of Inflammation* regarding helpfulness of peer reviewers’ comments was higher for open than for single-blind peer review, but did not reach significance.

### Limitations of this study

The RQI^12^ evaluates how detailed and thorough the reviewer reports are, but not whether the criticism is valid or whether the reviewers recognised the flaws in the manuscript. This kind of assessment requires specialist knowledge. A recent study of decision consistency in peer review of Post-doctoral Fellowship applications suggests that using as many as five reviewers per application may be optimal.^17^

We found only a moderate level of agreement between the raters of the quality of referee reports. Prior to rating the quality of reports for *BMC Microbiology* (single-blind peer review) and *BMC Infectious Diseases* (open peer review), formal discussions on how to use the RQI^12^ were not held. Subsequently, we found that agreement between the two independent raters was not high on individual questions, but there was moderate agreement for the overall mean score. Given this experience, before analysing reports for the *Journal of Inflammation*, the raters explicitly discussed the rating scale and agreed broadly on how they would score individual questions in general terms. Following this, we found improved rater agreement for all questions (see online supplementary table S1). However, the raters did not confer about the ratings for individual reports for any journal. The raters were not blinded to the identity of the reviewers. However, as the raters were not involved in managing peer review on these journals, the names of the reviewers were not familiar.

We were not able to use exactly the same research design for the *Journal of Inflammation* as used for *BMC Infectious Diseases* and *BMC Microbiology*. The *Journal of Inflammation* did not have a sufficient number of manuscripts reviewed by two referees, one of whom was suggested by the authors and one not. As a result, we did not have an equal number of reports from author- and non-author-suggested reviewers.

Another limitation of the study is the low number of author surveys available for the *Journal of Inflammation*. Although we analysed all available author surveys, it is possible that the lack of statistical significance of the results is due to the low number of available surveys rather than the lack of impact of peer review model.

We chose *BMC Microbiology* and *BMC Infectious Diseases* because the journals have a similar profile (table 1) and the same editorial processes, differing mainly in the peer review model. However, the journals do not have identical author bases and there remains the possibility that the differences we observed were due to some other factors despite our efforts to closely match the journals. Similarly, our results for the *Journal of Inflammation* may reflect temporal trends, such as the change of editorship noted above. To reduce the impact of such biases we selected articles sequentially by order of submission, starting from the date of change of review model.

## Conclusions

This study is the first to investigate the effect of author-suggested peer reviewers and openness of peer review, on the quality of peer review in the same journal and between very similar journals in the field of biology as well as medicine. We show that, in agreement with previous studies,^2^ ^13^ the quality of peer review reports in journals with open peer review is comparable with that of journals with single-blind peer review. Furthermore, open peer review improves constructiveness of peer reviewer comments.

Author-suggested reviewers tend to recommend acceptance more often than non-author-suggested reviewers, but the quality of peer review reports is similar regardless of the source of peer reviewer suggestions, which is also in agreement with previous reports.^1^ ^9^
